# Anthocyanins: The Infinite Properties of These Incredible Compounds

**DOI:** 10.3390/molecules28041812

**Published:** 2023-02-15

**Authors:** Pasquale Crupi

**Affiliations:** 1 Interdisciplinary Department of Medicine, University of Bari “Aldo Moro”, p.zza G. Cesare, 11-70124 Bari, Italy; pasquale.crupi@uniba.it or pasquale.crupi@crea.gov.it; Tel.: +39-347-125-2849; 2 CREA, Council for Agricultural Research and Economics, Research Center for Viticulture and Enology, Via Casamassima, 140-70010 Turi, Italy

Anthocyanins are acknowledged for their great heterogeneity of colors, from orange to blue hues in the visible spectrum. Due to their wide distribution in nature and structural diversity, they have made headlines. They display a large range of properties and perform diverse roles in plants and transformed products [1,2]. In recent decades, scholars have been turning their attention more and more insistently to these amazing molecules. Knowledge about their biosynthesis, bioactivities, and biological relevance, as well as their possible applications, is continuously broadening. Moreover, due to their relative abundance in diets and their chemical and biological versatility, they possess notable health-promoting features [3]. Nonetheless, we are only now truly beginning to understand how their absorption might relate to their bioactivity. Similarly, novel anthocyanin-enriching techniques are opening new opportunities for several applications in various industry sectors as food additives, cosmetics, and pharmaceuticals, for manufacturing food and non-food products. This Special Issue includes many highly advanced quality papers that focus on the synthesis, methods of analysis, bioavailability, anti-inflammatory and health promoting activity, and application in food and industry of these amazing compounds.

The manuscript by Pereira et al. [4] offers a valuable environmentally friendly, quick, and straightforward alternative to flavylium compounds’ challenging and labor-intensive functionalization, resulting in novel dyes with higher stabilities and dissimilar chromatic features. This work reports the functionalization of a pyranoflavylium pigment using 1-(3-dimethylaminopropyl)-3-ethylcarbodiimide hydrochloride coupling chemistry. Four cinnamic acids (i.e., 4-dimethylamino-, 4-amino-, 4-bromo-, and trans-cinnamic acids) were used to establish an ester bond with the hydroxyl group of the pyranoflavylium. An excellent reaction yields up to 99% were achieved by opportunely modulating the molar ratios, solvent, and reaction time. The structure of the functionalized pigments was fully clarified using one-dimensional (1H) and two-dimensional (COSY, HSQC, and HMBC) NMR experiments and HRSM analysis. Regardless of the type of functionalization, the UV-Visible spectrum showed a bathochromic shift (red region) on the maximum absorption wavelength and the absence of acid-base reactions throughout a broad pH range in comparison to the pyranoflavylium precursor.

As distiller grain is rich in natural active ingredients and can be used as an excellent antioxidant feed for goats, Lu et al. [5] study the feeding value of four different types of distiller grains (namely white, red, glutinous rice, and corn). Taken together, the results of their work showed that red and glutinous rice distiller grains could be used as protein feed; in particular, the former had higher levels of total phenols and total anthocyanins as well as DPPH scavenging activity. In addition, corn distiller grain might be considered as an alternative energy source feed, while white distiller grain exhibited higher total gas production.

Direct antioxidant activity and modulation of cell redox-dependent signaling are the main mechanisms associated with the beneficial properties of foods rich in anthocyanins against chronic inflammatory disorders such as intestinal bowel diseases. However, anthocyanins bioavailability is low due to their poor stability in the gastrointestinal tract. Therefore, Speciale et al. [6] performed an in vitro simulated gastrointestinal digestion of an anthocyanin-rich purified and standardized bilberry and blackcurrant extract (BBE), evaluating their composition by HPLC-DAD analysis and the antioxidant activity by FRAP assay, and studied the effects of a BBE gastrointestinal extract on Caco-2 exposed to TNF-α. The results confirmed the high instability of anthocyanins in the mild alkaline environment of the small intestine. However, the digested BBE maintained part of its bioactivity. Additionally, the BBE gastrointestinal extract inhibited the TNF-α-induced NF-κB pathway in Caco-2 and activated the Nrf2 pathway.

The review by Avula et al. [7] grouped various analytical methodologies on the characterization, quantification, and chemical profiling of the whole array of anthocyanins in berries and fruits within the last two decades. In addition, the factors affecting the stability of anthocyanins, including pH, light exposure, solvents, metal ions, and the presence of other substances, such as enzymes and proteins, were addressed. Several sources of anthocyanins, including berries and fruits with their botanical identity and respective yields of anthocyanins, were covered. In addition to chemical characterization, economically motivated adulteration of anthocyanin-rich fruits and berries due to increasing consumer demand are also the subject of discussion. Finally, the health benefits and the medicinal utilities of anthocyanins were briefly discussed.

Anthocyanins have been shown to be effective in chronic diseases because of their antioxidant and anti-inflammatory effects together with changes in the gut microbiota and modulation of neuropeptides such as insulin-like growth factor-1. Therefore, Panchal and Brown [8] examined whether these mechanisms may be effective to moderate the symptoms of disorders of the central nervous system in humans, including schizophrenia, Parkinson’s disease, Alzheimer’s disease, autism spectrum disorder, depression, anxiety, attention-deficit hyperactivity disorder, and epilepsy. Thus, anthocyanins from fruits and berries should be considered as complementary interventions to improve these chronic disorders.

In the review by Peniche-Pavía et al. [9], the authors focused on the importance of maize flavonoids in pigmentation and the human health sector. They included updated information about the enzymatic pathway of maize flavonoids, describing a total of twenty-one genes for the flavonoid pathway of maize: the first three genes participate in the general phenylpropanoid pathway, four genes are common biosynthetic early genes for flavonoids, and fourteen are specific genes for the flavonoid subgroups, the anthocyanins, and flavone C-glycosides. Then, they explained the tissue accumulation and regulation of flavonoids by environmental factors affecting the expression of the MYB-bHLH-WD40 (MBW) transcriptional complex. The study of transcription factors of the MBW complex is fundamental for understanding how the flavonoid profiles generate a palette of colors in plant tissues. Finally, they also included an update on the biological activities of cyanidin-3O-glucoside, the major maize anthocyanin, including anticancer, antidiabetic, and antioxidant effects, among others.

Finally, Alappat and Alappat [10] reviewed the biogenetics of anthocyanins together with their colors, structural modifications, and stability, along with their various applications in human health and welfare. In particular, they analyzed how research in food coloring, flavoring, and preserving industries has not satisfied the urge for natural and sustainable colors and supplemental products. The lability of anthocyanins under various formulated conditions is the primary reason for this delay. New gene editing technologies to modify anthocyanin structures in vivo and the structural modification of anthocyanin via semi-synthetic methods offer new opportunities in this area.

## Data Availability

Not applicable.
